# Literature review on hepatoprotective effects of diosgenin: possible mechanisms of action

**DOI:** 10.3389/fphar.2023.1226548

**Published:** 2023-09-12

**Authors:** Parvaneh Mohseni-Moghaddam, Manijeh Khanmohammadi, Mehrdad Roghani

**Affiliations:** ^1^ Department of Physiology, Faculty of Medicine, Tehran Medical Sciences, Islamic Azad University, Tehran, Iran; ^2^ School of Health and Biomedical Sciences, RMIT University, Bundoora, VIC, Australia; ^3^ Baker Heart and Diabetes Institute, Melbourne, VIC, Australia; ^4^ Neurophysiology Research Center, Shahed University, Tehran, Iran

**Keywords:** diosgenin, liver injury, fatty liver, liver fibrosis, diabetes mellitus, steatohepatitis

## Abstract

Liver diseases are among the major causes of death worldwide. Alcohol consumption, obesity, diabetes mellitus, viral infection, and drug-induced liver injury are common risk factors for the development of liver diseases. Diosgenin is a herbal steroidal sapogenin with hepatoprotective properties. This phytosteroid modulates lipid profile and prevents liver injury and fibrosis, metabolic associated fatty liver disease (MAFLD), steatohepatitis, and diabetes mellitus. Different mechanisms have been presented underlying the therapeutic properties of diosgenin. Diosgenin with antioxidant activity and ability to inhibit pro-inflammatory and apoptotic mediators as well as modulating gut microbiota is able to protect the liver. This literature overview summarizes the previously published studies regarding the hepatoprotective function of diosgenin against liver injury in different conditions with an emphasis on possible underlying mechanisms.

## Introduction

Liver diseases are responsible for almost two million deaths per year globally (Asrani et al., 2019). At present, liver disorders are considered as the 11th cause of mortality and at an accelerating pace for incidence (Griffin et al., 2023). Alcohol consumption, drugs, viral infection, diabetes mellitus and metabolic disorders, and obesity are known as predisposing factors for liver injury including metabolic associated fatty liver disease (MAFLD) and hepatocellular carcinoma (Devarbhavi et al., 2023; En Li Cho et al., 2023).

Natural products with comparable efficacy and safety and lower costs are regarded as good candidates and also as alternatives to provide liver protection against various illnesses including (MAFLD) (Ibrahim et al., 2023). Diosgenin (3β-Hydroxy-5-spirostene) is a phytosteroid sapogenin found in several plant families, such as Liliaceae*,* Dioscoreaceae*,* Scrophulariaceae*,* Solanaceae*,* Leguminosae*, Agavaceae,* Rhamnaceae*,* and Amaryllidaceae (Semwal et al., 2022). Diosgenin possesses numerous biochemical and physiological functions. The biochemical structure of diosgenin is analogous to cholesterol and other steroids, and accordingly, it is the main precursor of several pharmacologically active steroids, such as oral contraceptives and corticosteroids (Parama et al., 2020; Sun et al., 2021). Diosgenin has received a considerable amount of attention in recent years owing to its effectiveness in the treatment of numerous diseases, namely, cardiovascular diseases (Wang et al., 2018; Wu and Jiang, 2019), cancer (Sethi et al., 2018), neurological disorders (Cai et al., 2020), diabetes (Kalailingam et al., 2014), and hyperlipidemia (Li et al., 2019), in addition to its anti-inflammatory and antioxidant properties (Sethi et al., 2018). The current literature review compiles evidence on the hepatoprotective action of diosgenin with emphasis on potential involved mechanisms.

### Therapeutic potential of diosgenin in metabolic associated fatty liver disease (MAFLD)

MAFLD, formerly known as non-alcoholic fatty liver disease (NAFLD), is the most prevalent cause of liver injury worldwide (Younossi et al., 2016). NAFLD is a common liver injury distinguished by the accumulation of lipid droplets in the cytoplasm of the hepatocytes. This disease is a complex metabolic condition associated with genetic risk factors and lifestyle, ranging from simple steatosis, steatohepatitis, and fibrosis to cirrhosis (Matteoni et al., 1999; Targher, 2007; Than and Newsome, 2015; Karimi-Sales et al., 2018).

In a study conducted by Cheng et al., diosgenin was suggested as a potential agent to inhibit MAFLD (NAFLD) development by increasing the phosphorylation of adenosine monophosphate-activated protein kinase (AMPK) and acetyl-CoA carboxylase (ACC) and suppressing the expression of liver X receptor α (LXRα) in liver tissue. Diosgenin could also decrease mRNA expression of sterol regulatory element-binding protein-1c (SREBP-1c).

Another study indicated that diosgenin administration reduces excessive weight gain, serum triglyceride and total cholesterol levels, and fat deposition in the liver of MAFLD (NAFLD) rats. Diosgenin administration regulated the levels of bile acids, such as ursodeoxycholic acid 3-sulfate and lithocholic, and accordingly modulated the metabolism of lipids. Gut microbiota disorder observed in MAFLD (NAFLD) was another parameter restored by diosgenin. A significant correlation was also found between gut microbiota modulation and biomarkers of lipid and amino acid metabolism (Zhou et al., 2022).

Another research indicated that diosgenin ameliorates hepatosteatosis in obese mice. This phytosteroid modulated lipid profile, probably via down-regulating the gene expression of SREBP-1c and fatty acid synthase (FAS). Additionally, diosgenin, possibly through suppressing SREBP-1c and fatty acid synthase (FAS) expression, reduced lipid vacuoles in the hepatocytes and thus alleviated hepatic histological abnormalities. Diosgenin also decreased body weight gain and adjusted the serum liver enzyme levels, including ALT and aspartate aminotransferase (AST) (Khateeb et al., 2021).

In another study, diosgenin mitigated body weight, regulated lipid profile, and attenuated hepatosteatosis in animals with high-fat diet-induced hypercholesterolemia. The expression of Niemann-Pick C1-Like 1 (NPC1L1) and LXRα in the intestine and ATP-binding cassette (ABC) half-transporter ABCG5/G8 expression in both liver and intestine were investigated. The findings indicated that diosgenin may not only increase cholesterol excretion by increasing the expression of ABCG5/G8 in the liver and intestine but also prevent intestinal cholesterol absorption by down-regulating NPC1L1. LXRα expression was also decreased by diosgenin (Li et al., 2019). NPC1L1 is an essential transporter for cholesterol absorption (Altmann et al., 2004). The ABCG5/G8 complex inhibits the absorption of intestinal sterol by directly antagonizing the function of NPC1L1 (Brown and Yu, 2009; Dikkers and Tietge, 2010).

Based on pathological analyses, hepatosteatosis and intestinal structure were improved by diosgenin consumption. In diosgenin-fed rats, cholesterol 7α-hydroxylase or cytochrome P450 7A1 (CYP7A1), carboxylesterase-1 (CES-1), and scavenger receptor class B type I (SRB1) levels were increased in the liver. In contrast, farnesoid X receptor (FXR)-mediated signaling was inhibited, and these alterations contribute to the reduction of liver cholesterol. Diosgenin also inhibited intestinal CES-1 and increased SRB1, thereby promoting reverse cholesterol transport (RCT) and preventing intestinal cholesterol absorption. The results showed that diosgenin augmented the elimination of cholesterol through the SRB1/CES-1/CYP7A1/FXR pathway (Shi et al., 2021).

**FIGURE 1:**
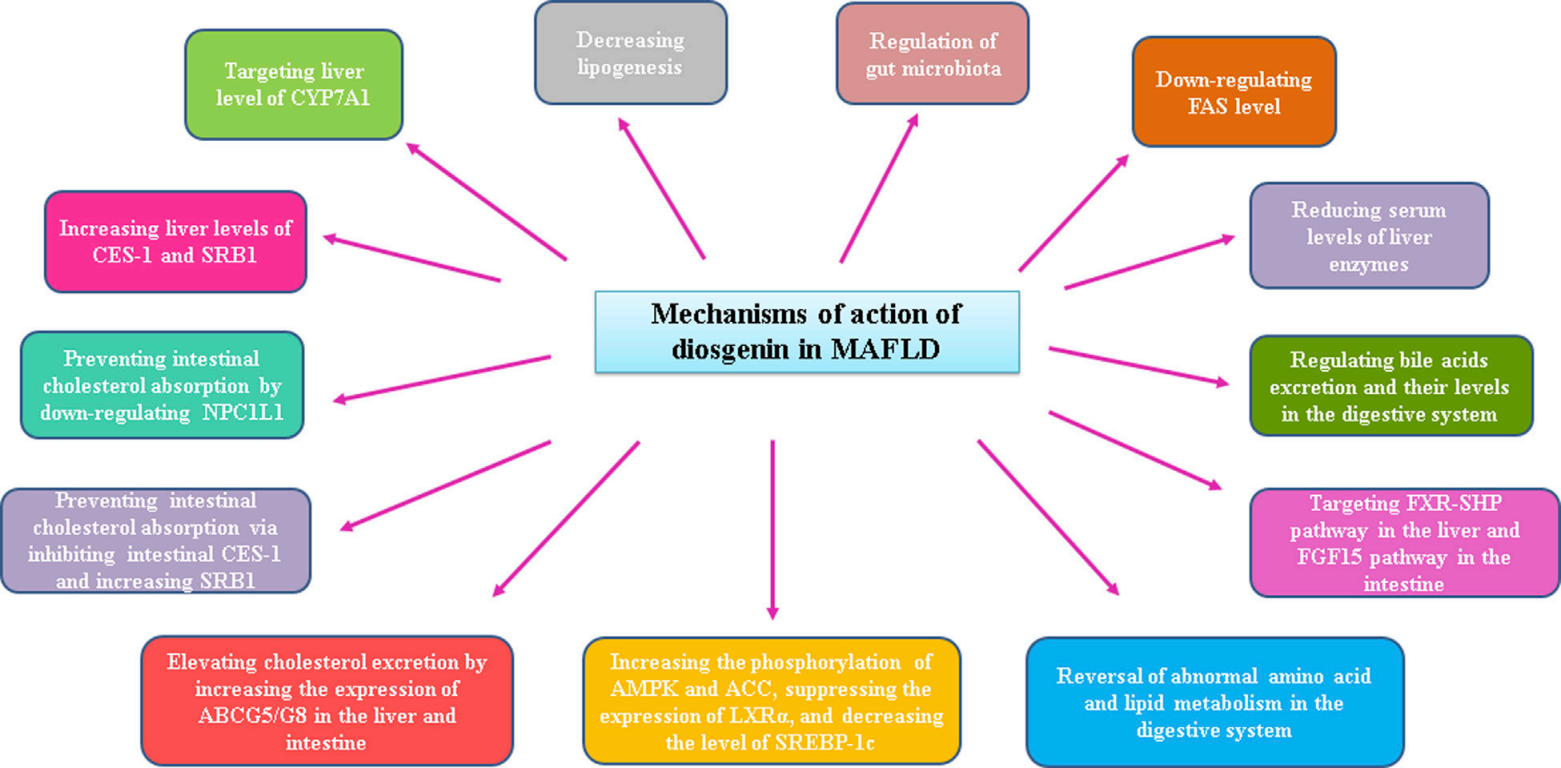
Overall modes of action of diosgenin in MAFLD.

In another study, administration of diosgenin resulted in a decrease in the levels of triglyceride, total cholesterol, total bile acid, ALT, and AST in both serum and liver, which ultimately caused an improvement in liver function. Yan and colleagues suggested that diosgenin has the potential to improve non-alcoholic steatohepatitis by influencing liver-gut circulation. Diosgenin causes bile acids excretion and modifies bile acids synthesis by regulation of gut microbiome such as Clostridia and also via targeting nuclear receptors including FXR and small heterodimer partner (SHP) in the liver and FXR-fibroblast growth factor 15 (FGF15) pathway in the intestine. Overall modes of action of diosgenin in MAFLD have been shown in Figure 1.

### Hepatoprotective effects of diosgenin in diabetes mellitus

Diabetes mellitus, as an increasing epidemic disease worldwide, is often associated with various disturbances in cardiovascular system and liver and kidney tissues. The liver is a major metabolic organ, and diabetes-associated metabolic disorders lead to hepatic dysfunctions, leading to a range of liver diseases, including fatty liver disease, cirrhosis, and hepatocellular carcinoma (Bedi et al., 2019).

In type 2 diabetes, insulin resistance index, dyslipidemia, and serum liver enzyme levels, such as ALT and AST, are decreased by diosgenin. Diosgenin augmented fatty acid *ß*-oxidation and prevented *de novo* lipogenesis via regulating AMPK-ACC/SREBP1 pathway. Carnitine palmitoyltransferase-1 (*CPT1*) and peroxisome proliferator-activated receptor α (*PPARα*) genes, two essential genes involved in fatty acid *ß*-oxidation, were significantly increased in diabetic rats treated with diosgenin. Endoplasmic reticulum (ER) stress is another important factor involved in diabetes-associated liver disorder. Diosgenin could inhibit ER stress through preventing protein kinase RNA-like endoplasmic reticulum kinase (PERK) and inositol-requiring enzyme-1 (IRE1) pathways (Zhong et al., 2022).

It has been reported that fenugreek seeds are the primary source of diosgenin (Sun et al., 2021). Fenugreek seed extract and diosgenin have been shown to exert hepatoprotective effects in diabetic rats by lowering serum levels of liver enzymes, liver levels of triglycerides, lipid peroxidation, and ER stress markers, while liver antioxidant and glycogen content were significantly increased. Histological analyses have also indicated that fenugreek seed extract and diosgenin alleviated liver damage associated with type two diabetes mellitus. The authors believe that these positive effects are mediated by normalizing oxidative stress and ER stress.

Results from another study indicated that diosgenin protects the liver from diabetic mice against cell injury, oxidative stress, lipid accumulation, and mild inflammation. Diosgenin also modulated the expressions of sirtuin 6 (SIRT6) and fatty acid transporters, such as fatty acid transport protein 2 (FATP2), cluster of differentiation 36 (CD36), and fatty acid binding protein 1 (FABP1). By application of the SIRT6 inhibitor (OSS-128167), the protective effects of diosgenin and also SIRT6 agonist (MDL800) were reversed. It seemed that diosgenin attenuates NAFLD in diabetic animals via inhibiting fatty acid uptake in the liver, which is mediated by SIRT6 upregulation (Nie et al., 2023).

**FIGURE 2:**
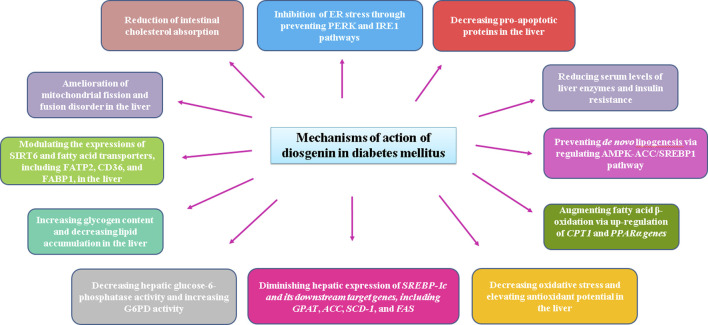
Modes of diosgenin effects under diabetic condition.

Supplementation of the diet of the diabetic animals with commercial diosgenin or yam steroidal sapogenin extract decreased the serum glucose level and hepatic glucose-6-phosphatase activity, while glucose-6-phosphate dehydrogenase (G6PD) activity was significantly increased in the liver compared to diabetic rats (McAnuff et al., 2005). Modes of diosgenin effects under diabetic conditions have been brought in Figure 2.

### Diosgenin effect against toxicant-induced liver injury and fibrosis

In liver injury induced by toxic chemicals, the hepatic metabolism of toxicants is pursued by free radical production and lipid peroxidation, causing liver damage (Mehendale, 2005; Karimi-Sales et al., 2018). Hepatic fibrosis is the hepatic tissue response to chronic injury, manifested by the synthesis and accumulation of extracellular matrix (ECM) without equivalent degradation (Karimi-Sales et al., 2018).

The effects of diosgenin against hepatotoxicity caused by carbon tetrachloride (CCL4) and cisplatin were evaluated. The results showed that liver fibrosis and liver function were ameliorated following diosgenin administration. Diosgenin decreased serum levels of liver enzymes, including ALT, AST, and alkaline phosphatase (ALP), as well as serum total bilirubin level. Liver content of hydroxyproline and ECM deposition, prominent markers of liver fibrosis, were also reduced by diosgenin. Diosgenin, probably via inhibiting the activation of hepatic stellate cells (HSCs), prevented liver fibrosis development (Nimbalkar et al., 2018).

In another study, performed on HSC-T6 cell line, transforming growth factor-β1 (TGF-β1)-induced HSC proliferation, TGF-β receptor I and II, collagen I, and α-smooth muscle actin (α-SMA) expressions were decreased following treatment with diosgenin. Diosgenin also inhibited the phosphorylation of Smad3 induced by TGF-β1 signaling.

In lipopolysaccharide (LPS)-D-galactosamine-induced acute liver failure, diosgenin reduced serum liver enzyme levels and hepatic levels of ROS, malondialdehyde (MDA), and inflammatory mediators, such as tumor necrosis factor-α (TNF-α), interleukin-1β (IL-1β), IL-6, nuclear factor-kappa B (NF-κB), toll-like receptor 4 (TLR4), and mitogen-activated protein kinases (MAPK), in addition to a reduction in the activity of myeloperoxidase, as a marker of neutrophil infiltration. Besides, diosgenin increased the *hepatic level of nuclear factor erythroid 2-related factor 2* (Nrf2) and SOD activity. The hepatoprotective activity of diosgenin against liver injury was attributed to the inhibition of oxidative stress, inflammation, and neutrophil infiltration (Mohamadi-Zarch et al., 2020). Inflammation is a prevalent trigger of liver diseases and is known to be the main cause of liver injury, leading to the progression of NAFLD to severe liver fibrosis and, ultimately, to hepatocellular carcinoma (Del Campo et al., 2018).

In a rat model of liver injury caused by methotrexate, diosgenin reduced neutrophil infiltration and improved mitochondrial health. It seems that methotrexate-mediated oxidative stress causes a decrease in mitochondrial membrane potential, and diosgenin could restore this reduction (Haddadzadeh Niri et al., 2022).

**FIGURE 3:**
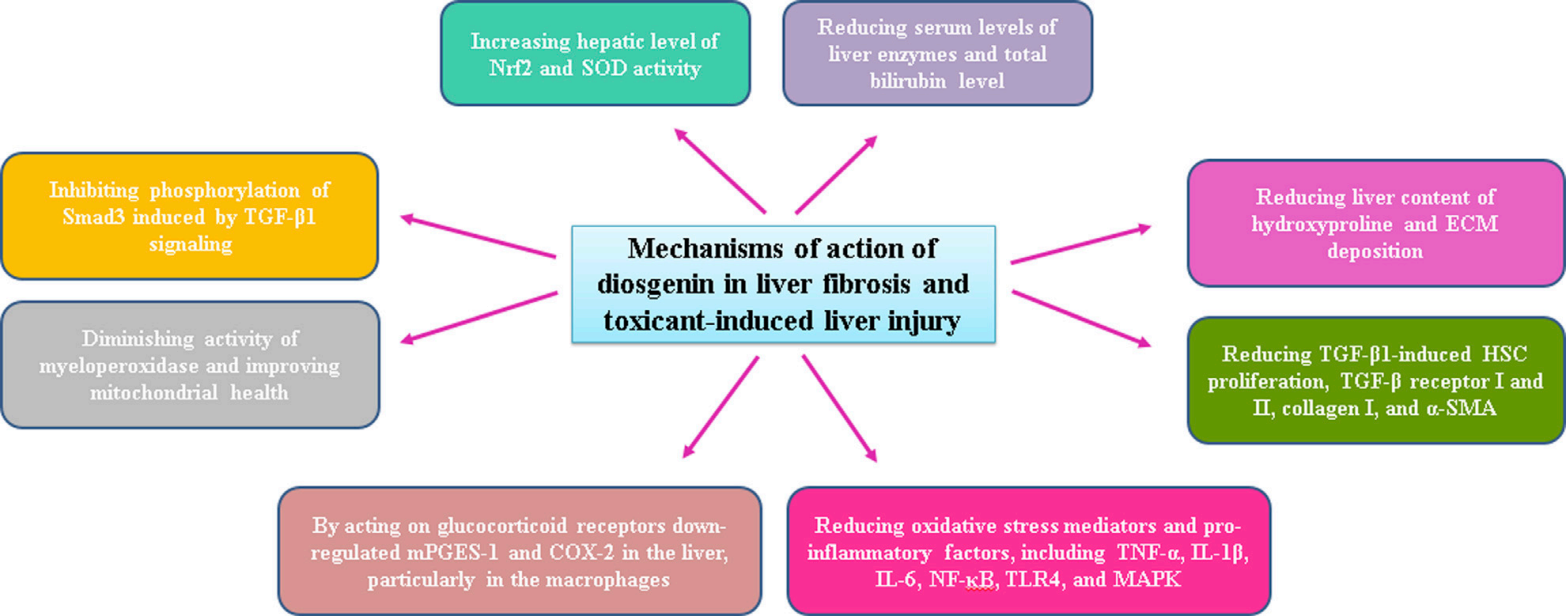
Possible mechanisms of diosgenin action following toxin-induced hepatotoxicity.

In LPS-induced liver injury in mice, treatment with diosgenin downregulated microsomal prostaglandin E synthase-1 (mPGES-1) and cyclooxygenase-2 (COX-2) in the liver, particularly in the macrophages. It has been reported that diosgenin, by acting on glucocorticoid receptors, has caused a decrease in the expression of the mentioned molecules (Tsukayama et al., 2021). Possible mechanisms of diosgenin action following induction of toxin-induced hepatotoxicity have been presented in Figure 3.

### Promising beneficial effects of diosgenin in other pathologic conditions

In a rat model of chronic renal failure caused by adenine containing diet, the potential hepatoprotective role of diosgenin was shown through increasing antioxidant capacity and inhibiting protein structural damage in the liver (Manivannan et al., 2013). Protein structural damage can occur in response to the overproduction of free radicals and increased urea-induced toxicity (Sokołowska et al., 2011).

In animals on an atherogenic diet, diosgenin reduced inflammatory mediators, including TNF-α, COX-2, and NF-κB, in different tissues, such as the liver, heart, and brain. Serum liver enzyme levels and steatosis in the liver were also alleviated following treatment with diosgenin (Binesh et al., 2018).

Treatment of liver cancer is generally mediated by chemotherapy, whereas chemotherapeutic drugs, such as doxorubicin, may result in drug resistance and toxicity. The targeted delivery of chemotherapeutic drugs in combination with natural products is beneficial to improve the malignant tumor along with increased efficiency and lowered toxicity. In a study, a diosgenin-based liposome loaded with doxorubicin was formed to evaluate their synergistic effects against liver cancer and the results were compared with a group of animals that received commercial doxorubicin liposome (cholesterol-based liposomes). Diosgenin not only replaced cholesterol as a regulator of the membrane to maintain liposome stability but also served as an adjuvant to doxorubicin for synergistic treatment. In comparison with commercial doxorubicin liposome, diosgenin-based liposome loaded with doxorubicin was more efficient to treat tumor by inhibition of the tumor cell proliferation, induction of the apoptosis and less toxicity (Chen et al., 2022).

## Conclusion

**FIGURE 4:**
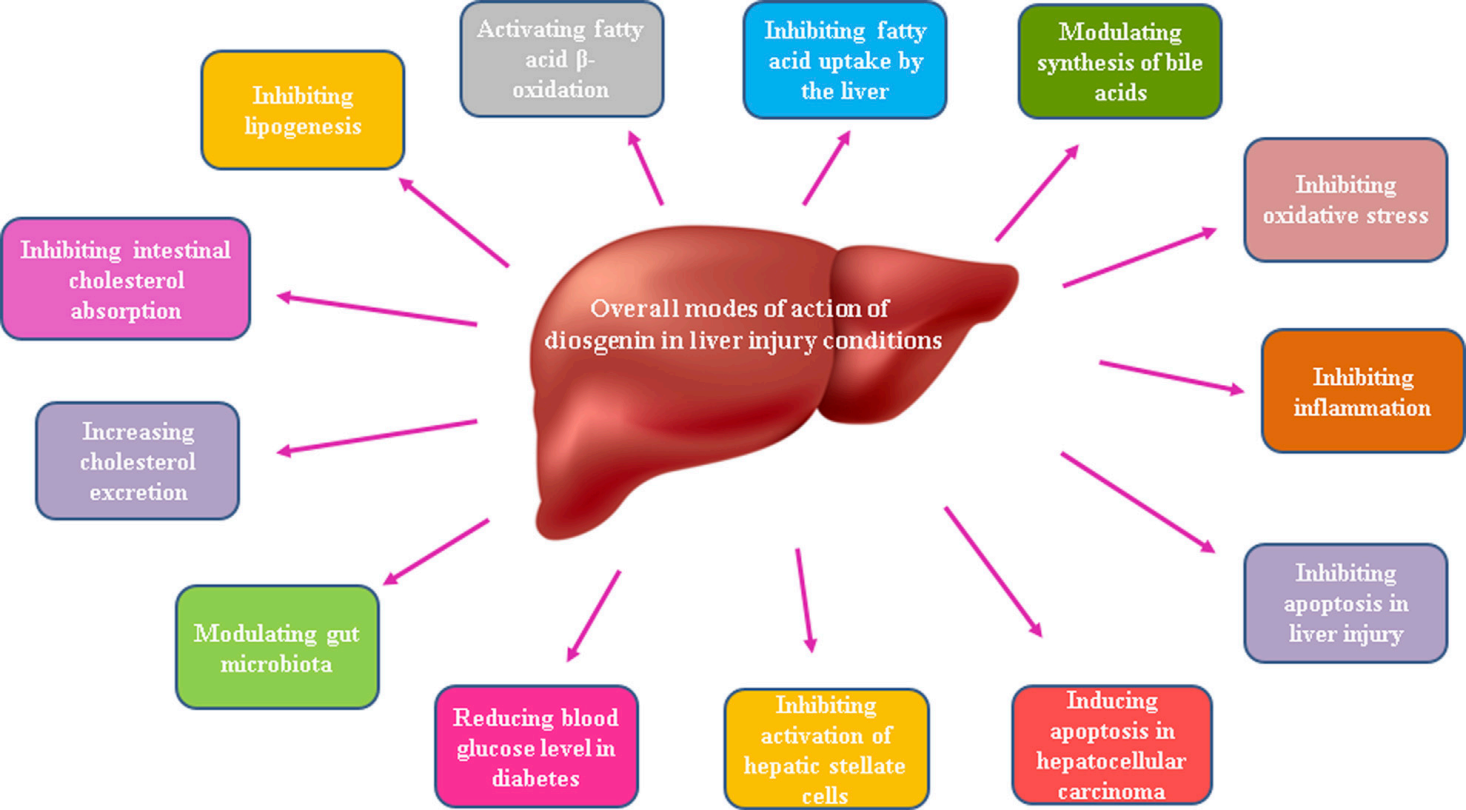
Possible hepatoprotective mechanisms of diosgenin in liver injury conditions.

Existing research evidence shows promising hepatoprotective effects of the phytosteroid diosgenin in various pathologic conditions including non-alcoholic fatty liver disease, hepatosteatosis, diabetes mellitus, liver fibrosis, and even hepatocellular carcinoma. Beneficial effects of this phytosteroid are mainly attributed to its ability to enhance antioxidant system and to attenuate oxidative stress besides its inhibition of pro-inflammatory and pro-apoptotic mediators and also modulation of gut microbiota. Possible hepatoprotective mechanisms of diosgenin in liver injury conditions have been indicated in Figure 4.
